# Early maternal care restores LINE-1 methylation and enhances neurodevelopment in preterm infants

**DOI:** 10.1186/s12916-020-01896-0

**Published:** 2021-02-05

**Authors:** Camilla Fontana, Federica Marasca, Livia Provitera, Sara Mancinelli, Nicola Pesenti, Shruti Sinha, Sofia Passera, Sergio Abrignani, Fabio Mosca, Simona Lodato, Beatrice Bodega, Monica Fumagalli

**Affiliations:** 1 Department of Clinical Sciences and Community Health, University of Milan, Milan, Italy; 2 Istituto Nazionale di Genetica Molecolare “Enrica e Romeo Invernizzi” (INGM), Milan, Italy; 3 Fondazione IRCCS Ca’ Granda Ospedale Maggiore Policlinico, NICU, Milan, Italy; 4 Department of Biomedical Sciences, Humanitas University, Pieve Emanuele, Milan, Italy; 5 IRCCS Humanitas Clinical and Research Center, Rozzano, Milan, Italy; 6 Department of Statistics and Quantitative Methods, Division of Biostatistics, Epidemiology and Public Health, University of Milano-Bicocca, Milan, Italy

**Keywords:** Prematurity, Early maternal care, LINE1 methylation, Neurodevelopment

## Abstract

**Background:**

Preterm birth affects almost 9–11% of newborns and is one of the leading causes of childhood neurodevelopmental disabilities; the underlying molecular networks are poorly defined. In neurons, retrotransposons LINE-1 (L1) are an active source of genomic mosaicism that is deregulated in several neurological disorders; early life experience has been shown to regulate L1 activity in mice.

**Methods:**

Very preterm infants were randomized to receive standard care or early intervention. L1 methylation was measured at birth and at hospital discharge. At 12 and 36 months, infants’ neurodevelopment was evaluated with the Griffiths Scales. L1 methylation and CNVs were measured in mouse brain areas at embryonic and postnatal stages.

**Results:**

Here we report that L1 promoter is hypomethylated in preterm infants at birth and that an early intervention program, based on enhanced maternal care and positive multisensory stimulation, restores L1 methylation levels comparable to healthy newborns and ameliorates neurodevelopment in childhood. We further show that L1 activity is fine-tuned in the perinatal mouse brain, suggesting a sensitive and vulnerable window for the L1 epigenetic setting.

**Conclusions:**

Our results open the field on the inspection of L1 activity as a novel molecular and predictive approach to infants’ prematurity-related neurodevelopmental outcomes.

**Trial registration:**

ClinicalTrial.gov (NCT02983513). Registered on 6 December 2016, retrospectively registered.

**Supplementary Information:**

The online version contains supplementary material available at 10.1186/s12916-020-01896-0.

## Background

Prematurity, which is defined as birth before 37 weeks of gestation, affects 9–11% of neonates globally, and it is the second leading cause of death in children below 5 years of age and the most important in the first month of life [1, 2]. Among preterm infants, about 16% are born very preterm (< 32 weeks of gestation) [2]. This condition is associated with a considerable risk to develop acute and chronic postnatal morbidities and long-term neurodevelopmental disabilities [3]. Indeed, up to 15% of very preterm infants suffer from severe neurologic disorders, mainly related to the occurrence of acquired brain lesions [4], and up to 50% of preterm infants experience other neurocognitive impairments in different areas of development (e.g., language, behavior, visual processing, academic performances, and executive functions) [5] or neuropsychological problems, including attention deficit/hyperactivity disorder (ADHD) or autism spectrum disorders (ASD) [6, 7].

Besides the documented role played by gestational age (GA) at birth and by the occurrence and severity of postnatal morbidities, the impact of early exposure to the hazards of extrauterine life has been recently emphasized. Indeed, during their stay in neonatal intensive care unit (NICU), preterm infants face early environmental stress, mainly represented by deprivation of physiological intrauterine sensory experiences [8–10], excessive, potentially harmful, neurosensory stimulation, and prolonged separation from their parents [11]. Overall, these environmental stressors act in critical a window for the developing brain (corresponding to the last trimester of pregnancy or early postnatal life in case of premature birth) [12], in which cognitive and emotional processing development relies on the proper assembly of cerebral cortex circuits (which includes both the neocortex and the hippocampus) [13–16]. Alterations of such developmental programs have been associated to prominent long-term consequences in childhood and adulthood [17–19].

In this framework, developmental care, conceived as a strategy to reduce NICU stressful factors and promote maternal engagement, has been demonstrated to improve brain maturation, as assessed by magnetic resonance imaging (MRI), and neurodevelopmental outcomes [20, 21]. Interestingly, recent findings have highlighted the crucial role of maternal care on modulating the detrimental effects of early life exposure [22]. Based on these observations, more active and modulated stimulations during NICU stay have been recently proposed; these early intervention strategies, through the enhancement of sensory experiences, as provided by infant massage or maternal voice or music listening, could contribute to promote infants’ neurobehavior and brain development [23, 24]. How these early life experiences can modulate at molecular level the brain development and, ultimately, child’s behavior is still an unsolved issue.

Mobile DNA elements have the ability to change their genomic position, either by a DNA-based (transposition) or RNA-based (retrotransposition) mechanism. Retrotransposition is one of the main forms of somatic mosaicism in the brain [25]. Among Transposable Elements (TEs), LINE-1 (L1), which cover about 18% of the human genome [26], have been extensively described to retrotranspose in neurons from fly to humans [27–29], a mechanism taking place during neural progenitor development and differentiation [30, 31]. L1 can move to different locations (de novo insertion sites) by the activity of a reverse transcriptase (RTase), encoded by L1 itself, which reverse-transcribes and integrates a L1 cDNA copy, which is usually 5′ end truncated [26]. L1 activity is finely modulated at the level of its endogenous promoter, where a CpG island demethylation is associated with L1 somatic mobilization in the brain [32]. The deregulation of L1 activity has been described in neuronal models of debilitating neurological diseases as Rett syndrome [32], schizophrenia [33], autism spectrum disorder [34], and bipolar and major depressive disorder [35, 36]. Notably, early life experience and maternal deprivation has been reported to drive variability in L1 methylation and copy number variations (CNVs) within the mouse hippocampal neuronal genome of the progeny, influencing their behavior [37].

In the current study, we sought to document for the first time in humans whether enhanced maternal care could modulate neurodevelopment in preterm infants and whether this clinical aspect could be reflected by L1 methylation levels in perinatal stages. In order to precisely assess the perinatal window in which L1 epigenetic setting is established in the brain, we leveraged on comparative analysis in developing mouse brain and further dissected L1 dynamics (methylation and CNVs).

## Methods

### Study cohort

All the preterm infants consecutively born between 25^+0^ and 29^+6^ weeks of gestational age (GA) at the same institution were eligible. Exclusion criteria include multiple pregnancy (triplets or higher), genetic syndromes and/or malformations, and infants who developed severe neonatal comorbidities including severe brain lesions. Inclusion and exclusion criteria were designed to enroll a homogeneous cohort of preterm infants. The full study protocol is described in [38]. Adherence to the early intervention protocol was required and documented in a parental self-report diary.

### Study design

Infants were randomly assigned either to receive (i) standard care or (ii) an additional early intervention protocol based on maternal care. Standard care, according to the routine clinical protocol of the NICU, included kangaroo mother care, minimal handling, and non-pharmacological pain management. The early intervention protocol included, over routine clinical care, the PremieStart [39], which is based on parental involvement, and enriched multisensory stimulation proposed by parents after a period of training. This intervention included both tactile stimulations, through infant massages performed twice a day, and visual interaction provided at least once a day with a black and white toy or parents’ face. A complete detailed description of the intervention is available in [38].

The randomization was performed using sealed envelopes prepared in groups of 10 through computer-generated randomization. The randomization sequence was concealed until the group allocation was assigned, and the examiners (both biologist and psychologist that performed the follow-up examination) remained blinded for the entire study period.

The present study is a post hoc analysis of a larger randomized controlled trial (RCT) that included 70 very preterm infants born between 25^+0^ and 29^+6^ weeks of gestational age (GA), recruited between April 2014 and January 2017. The trial aimed at assessing the effectiveness of an early intervention program, based on early parental involvement in neonatal care, in promoting visual function and neurodevelopment in preterm infants. A positive effect of early intervention on visual function maturation [40] (as primary outcome) and on full oral feeding acquisition (as short-term secondary outcome) was demonstrated [38]. Within this context, exploratory analysis has been performed in a sub-group of infants to investigate the effect of preterm birth and early interventions on L1 modulation. The sub-group of infants included for L1 methylation analyses is representative of the overall cohort enrolled in the larger RCT.

### Sample collection

In preterm infants, cord blood samples were collected at birth and peripheral blood samples were harvested at hospital discharge (around term equivalent age (TEA)). Peripheral blood was obtained during blood sampling performed for routine blood examination, according to clinical practice. In healthy full-term infants’ cord blood samples were collected at birth only in infants born by cesarean section after uneventful pregnancies. Each sample consisted of 0.5 mL of cord/peripheral blood.

### FACS analysis and isolation of granulocytes and lymphocytes populations

Fresh cord blood samples derived from full-term and preterm infants were subjected to erythrocytes lysis following the manufacturer’s instruction (BD lysis buffer). Nucleated blood cells were then stained with anti CD45 for 30 min at 37 °C, different subpopulations were identified gating on CD45 and SSC as described in [41, 42]. Most abundant populations as granulocytes and lymphocytes were then sorted to be further subjected to DNA methylation analysis. Granulocytes were sorted as the population CD45 high with the highest SSC while lymphocytes were sorted as the population CD45 high with the lowest SSC.

### Neurodevelopmental assessment

Neurodevelopment was assessed as a post hoc analysis of the larger RCT. At 12 months corrected age and at 36 months chronological age the preterm infants underwent the Griffiths Scales to assess neurodevelopment (the Griffiths Mental Development Scales (GMDS-R) [43] at 12 months corrected age and its updated version (Griffiths-III) at 36 months [44]). These evaluations comprise five subscales (score range 50–150): locomotor, personal-social, hearing and language, eye and hand coordination, and performance (named Foundation of Learning in the Griffiths-III). Standardized scores are defined as 100 ± 12 (mean ± SD) for the general quotient and 100 ± 16 (mean ± SD) for each domain in the GMDS-R; 100 ± 15 (mean ± SD) for both the general quotient and the subscales in the Griffiths-III. For both scales, a standardized score > 2 SD below the mean indicates severe impairment, and a standardized score > 1 SD below the mean indicates mild impairment.

### Animals

CD-1 mice were housed under controlled conditions for temperature and humidity, using a 12:12-h light-dark cycle. Mice were mated overnight, and females were separated the following morning and checked for vaginal plugs (embryonic day, E 0.5). CD-1 animals deliver pups between day E19 and E20. Cesarean sections (C-secs) were performed at embryonic days E15.5, E18.5, and P0. Pups were sacrificed by decapitation at different time points: at embryonic day E15.5, E18.5, and at postnatal day P0, P3, and P14. At each developmental stage, 4 mice were sacrificed and brains collected to manually microdissect hippocampal, cortical, and cerebellar tissue under a stereomicroscope in sterile conditions. From the same mice, also blood samples were collected at E18.5 and at postnatal day P0, P3, and P14. We excluded E15.5 from blood samples given the low amount of material that was not sufficient for the subsequent molecular analysis; we excluded one sample at P0 because not usable. Blood and microdissected tissues were store at − 80 °C until gDNA extraction was performed.

### DNA extraction

Genomic DNA was isolated with standard phenol-chloroform extraction techniques from human whole cord or peripheral blood that is all the circulating nucleated cells; genomic DNA was isolated from mouse blood, hippocampus, cortex, and cerebellum and was isolated with standard phenol-chloroform extraction techniques.

### Bisulfite conversion

Five hundred nanograms of genomic DNA from each sample were bisulfite-treated using the MethylEdge™ Bisulfite Conversion System (Promega, Madison, USA) following the manufacturer’s protocol.

### Methylation assay in human samples

The methylation analysis of CpG island within the human L1 promoter was conducted as reported in [31] with minor modifications. The primer sequences are the following:

- hL1-5′UTR For: 5′ - AAGGGGTTAGGGAGTTTTTTT – 3′
- hL1-5′UTR Rev: 5′ - TATCTATACCCTACCCCCAAAA – 3′

In each PCR, 40 ng of bisulfite-converted DNA were combined with primers at 0.5 μM final concentration and GoTaq™ Hot Start Green Master Mix (Promega) in a final volume of 50 μL. PCR conditions were as follows: 95 °C for 2 min followed by 30 cycles of 95 °C for 45 s, 56 °C for 1 min and 72 °C for 30 s, followed by a final step of 72 °C hold for 4 min.

The product of amplification is 363 bp of length and contains 19 CpGs. The resulting PCR products were checked by agarose gel electrophoresis and then purified by PureLink™ Quick Gel Extraction & PCR Purification Combo Kit (Invitrogen-Thermo Fisher Scientific, USA). They were then cloned into pGEM-T Easy Vector System I (Promega) using a molar ratio insert: vector of 6:1. Sanger sequencing was performed by GATC Biotech, using the reverse sequencing primer pGEM Seq Rev: 5′-GACCATGATTACGCCAAGCTA – 3′. Resulting chromatograms were examined for sequencing quality using FinchTV software. At least 10 sequenced clones per sample were analyzed in Fig. 3 and Additional file 1: Fig. S1, as suggested in [45].

### Analysis of Sanger sequencing in human samples

To analyze the conversion efficiency and the methylation status of the CpG sites, FASTAQ files were analyzed by QUMA (*QUantification tool for Methylation Analysis*) software (CDB, Riken, Japan) [46]. For the L1 promoter methylation, we excluded from the analysis three (CpG 2, 6, and 9) of the 19 CpGs due to the high degree of variability among the analyzed sequences compared to the consensus sequence used (L19092.1 Human LINE1 (L1.4)). Sequences with a > 90% of cytosine residues converted were used for subsequent analysis. Total percent methylation was calculated as the number of methylated CpGs divided by the number of total CpGs (both methylated and unmethylated) multiplied by 100. To determine the methylation status of each CpG site, we calculated the percentage of methylation of each CpG site as the number of methylation events at a specific CpG site divided by the total number of sequenced and analyzed clones.

### Methylation assay in mouse samples

The methylation analysis of CpG island within the murine L1MdTf monomer and IAPLTR1a were conducted as reported in [37] with minor modifications. Given the peculiar monomeric and highly repeated nature of the mouse L15′UTR, we performed this methylation analysis with a Next Generation sequencing approach. A detailed list of primer sequences used for the amplification is reported in Additional file 2: Table S3. Briefly, both for L1MdTf monomer and IAPLTR1a, we used primers with Illumina barcode index (Illumina Truseq LT 6-mer indices): each organ in each developmental stage was associated to a distinct couple of Forw and Rev primers 5′ - end tagged, in order to be unambiguously identified in the sequencing analysis step (see Additional file 2: Table S3).

Each PCR was performed with 16–40 ng of bisulfite-converted DNA were combined with primers at 0.5 μM final concentration and GoTaq™ Hot Start Green Master Mix (Promega) in a final volume of 50 μL. PCR conditions were as follows: 95 °C for 2 min followed by 30 cycles of 95 °C for 45 s; 56 °C for 1 min and 72 °C for 5 s, followed by a final step of 72 °C hold for 4 min.

For L1MdTf monomer the product of amplification is 191 bp of length and contains 13 CpGs while for IAPLTR1a the product of amplification is 205 bp of length and contains 10 CpGs. The resulting PCR products were checked by agarose gel electrophoresis and then purified by Agencourt AMPure XP beads (Beckman Coulter) according to the manufacturer’s instructions. DNA concentration was quantified using a Qubit dsDNA HS Assay kit. All the L1MdTf and IAPLTR1a amplicons were then pooled in equimolar quantities to obtain a final pooling concentration of 2 ng/μL. Library for DNA sequencing was produced on the pooled PCRs. Paired-end 2 × 150 bp sequencing was performed on a HiSeq platform (Illumina) by Eurofins GATC Biotech.

### Analysis of NGS sequencing in mouse samples

A total of 22,481,000 reads were obtained from bisulfite sequencing and were assigned to samples based on the primers with Illumina barcode index. Briefly, no mismatch was allowed for the barcode index and a maximum of 5 mismatches were allowed for the target primer. None of the reads assigned to IAPLTR1a target aligned on L1MdTf and vice versa. Prior to mapping, reads were trimmed for low quality using Trimmomatic [47] (parameters: ILLUMINACLIP:TruSeq3-PE.fa:2:30:10 LEADING:3 TRAILING:3 SLIDINGWINDOW:4:15 MINLEN:75). 15,368,000 reads were obtained post trimming with an average of 80,000 reads for each sample. The paired reads were mapped using Bismark [48] (parameters: --local -N 1 -L 15 --non_directional) with an average mapping efficiency 99.05%. DNA methylation data was called using MethylDackel (https://github.com/dpryan79/MethylDackel). Sample correlation analysis was performed using methylKit [49] and all biological replicates of a given organ within a developmental stage showed a correlation higher than 95%. Methylation analysis for L1MdTf was focused on the YY1 binding site, corresponding to the CpG sites 8 and 9, as reported by [37] and on four CpG sites for IAPLTR1a, as reported by [37]. Briefly, the methylation status of each CpG site was calculated as the number of methylation events at a specific CpG site divided by the total number of analyzed sequences. Sample methylation level was calculated as the number of methylated CpGs divided by the number of total CpGs (both methylated and unmethylated) multiplied by 100.

### TaqMan PCR for L1 expression and CNVs analysis

For L1 CNVs, 300 ng of genomic DNA was treated with Exonuclease I, following the manufacturer’s instructions (40 U of Exonuclease I in reaction buffer (67 mM glycine-KOH (pH 9.5 at 25 °C), 67 mM MgCl_2_, 1 mM DTT) were used at 37 °C for 30 min and inactivated at 85 °C for 15 min). Efficiency of digestion was proved on 300 ng of gDNA pooled with 300 ng of a 120 bp ssDNA oligonucleotide (Additional file 1: Fig. S3 c). Digested DNA was further subjected to phenol-chloroform purification. Extracted DNA was quantified using Qubit HS DNA kit (Invitrogen) and diluted to a concentration of 80 pg/μL and used for subsequent experiments.

Quantitative PCR experiments were performed on a StepOne Plus (Thermo Fisher Scientific) with minor modifications to the method reported in [31]. In each multiplexed PCR, two TaqMan probes, labeled FAM and VIC, were combined; 80 pg of genomic DNA was combined with gene-specific primers, TaqMan-MGB probes, and 10 μL of iQ multiplex PowerMix (Biorad) in a total volume of 20 μL. Primers’ concentration was 0.4 μM and TaqMan probes’ concentration 0.4 μM. PCR conditions were as follows: 95 °C for 2 min followed by 40 cycles of 95 °C for 10 s and 59 °C for 60 s.

Standard curves of genomic DNA ranging from 200 ng to 0.2 ng were performed to verify that the 80 pg dilution was within the linear range of the reaction. For CNVs, the quantification includes from five to eight technical replicates. For assays on mouse genome, we adapted a TaqMan probe for the same amplicon reported in [32, 37]. Probes’ and primers’ sequences are reported below:

- mL1 ORF2 F: 5′ – CTGGCGAGGATGTGGAGAA - 3′
- mL1 ORF2 R: 5′ – CCTGCAATCCCACCAACAT - 3′
- mL1 ORF2 Taqman probe: 5′ – TGGAGAAAGAGGAACACTCCTCC - 3′
- mL1 5S F: 5′ – ACGGCCATACCACCCTGAAC - 3′
- mL1 5S R: 5′ – AGCCTACAGCACCCGGTATTC - 3′
- mL1 5S Taqman probe: 5′ – GATCTCGTCTGATCTCGGAAGCTAAG - 3′

### Study approval

The present study was approved by the Ethics Committee Milano Area B. The trial is registered at ClinicalTrial.gov (NCT02983513). Written informed consent was signed by both parents before inclusion in the study (both for preterm and full-term infants). All experimental procedures were performed in compliance with national and EU legislation, and Humanitas Clinical and Research Center, approved by the Animal Care and Use Committee (6B2B3.N.8EK).

### Accession number

The data from bisulfite sequencing has been submitted in NCBI GEO (GSE136844).

### Statistical analysis

The present study reports the results of a post hoc analysis of a larger randomized controlled trial (NCT02983513). The power calculation and sample size analysis were performed according to the primary outcome aimed at assessing the effectiveness of an early intervention program in enhancing visual function as a short-term neurodevelopmental outcome in very preterm infants [40]. Demographic and baseline characteristics were described as mean ± SD, median and range or number and percentage. Independent *t* test and Mann-Whitney *U* test were used in the comparison of continuous variables with normal distribution and non-normal distribution respectively. For the comparison of qualitative data, Fisher’s exact test was used. Shapiro-Wilk test was used to test the normal distribution of the data. To assess the differences between full-term and preterm infants, and between treatment groups in total L1 methylation and on each CpG, unpaired *t* test and two-way ANOVA model with Tukey’s HSD post hoc tests were used. Linear regression model was used to study the relationship between L1 methylation at NICU discharge and the intensity of care (mean number of massages per week) and independent *t* test and Mann-Whitney *U* test were used to assess the difference in neurodevelopmental outcome between standard care and early intervention groups at 12 and 36 months.

Mouse brain regions’ methylation at different stages of development were analyzed using one-way ANOVA and Tukey’s HSD post hoc tests. All tests were two-tailed, and *p* < 0.05 was considered significant for all tests. Statistical analyses were performed using R version 3.5.3 (R Foundation for Statistical Computing, Vienna, Austria).

## Results

### Characteristics of study participants

**Fig. 1:**
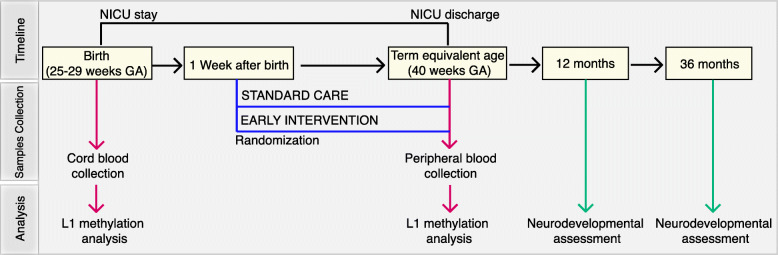
Timeline of the study. Preterm infants born between 25^+0^ and 29^+6^ weeks gestational age (GA) were recruited. At birth, cord blood samples were collected. One week after birth, preterm infants were randomized to either receive standard care or early intervention during NICU stay. At term equivalent age (40 weeks GA), before NICU discharge, peripheral blood samples were harvested. At 12 months corrected age and at 36 months chronological age neurodevelopmental assessment was performed. L1 promoter methylation analysis was performed on genomic DNA extracted from cord blood and peripheral blood

**Fig. 2:**
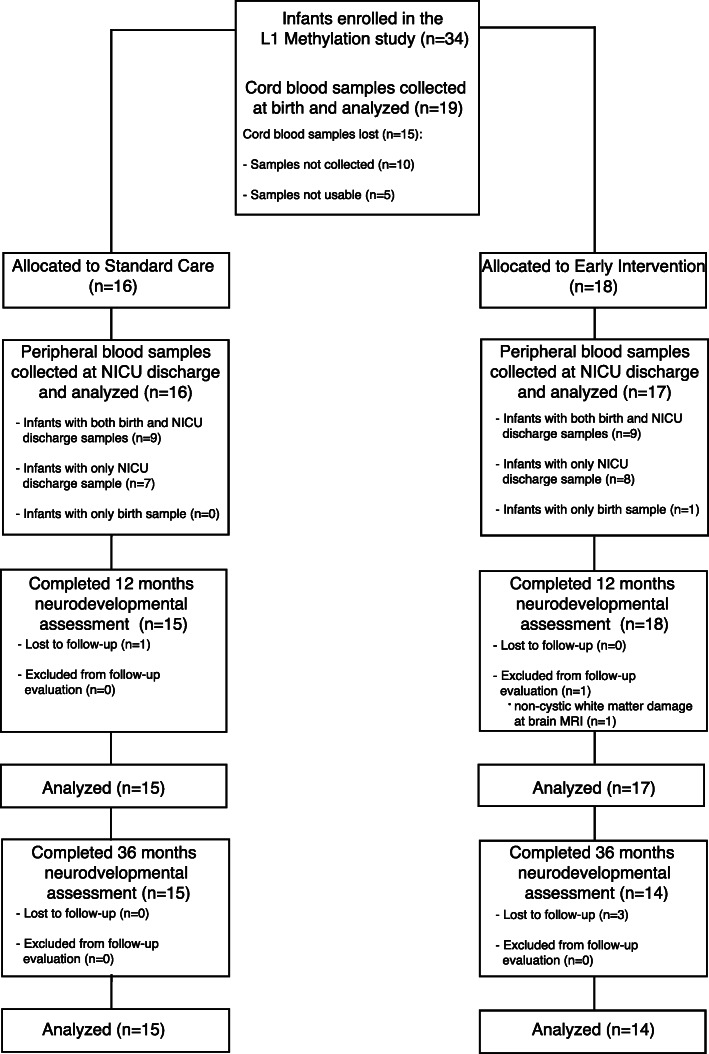
Flow chart of the study. CONSORT flow diagram showing patient enrollment, allocation to standard care and early intervention groups, blood samples collection, subsequent L1 promoter methylation analysis, and neurodevelopmental evaluation

To investigate the effects of preterm birth and early interventions on L1 modulation and neurodevelopmental outcomes, a post hoc analysis was conducted on 34 very preterm infants born between 25^+0^ and 29^+6^ weeks of gestational age (GA). A schematic representation of the study timeline, sample collection, and of the molecular and clinical analysis performed is provided in Fig. 1. Among the 34 infants, 19 cord blood samples were collected at birth (Fig. 2).

One week after birth and only in condition of clinical stability, all the enrolled infants were randomized to either receive standard care or early intervention (Fig. 1). Standard care, in line with NICU routine care protocols, included kangaroo mother care and minimal handling. Early intervention, in addition to routine care, included a parental training program together with enriched multisensory stimulation (infant massage and visual interaction, see the “Methods” section) promoted by parents as fully described in [38]. A daily diary was given to parents to record all interventions performed and to retrospectively quantify the effects of maternal care and multisensory stimulation. The study was conducted in a NICU with open access to parents (24 h a day for 7 days a week).

At NICU discharge peripheral blood samples were collected, named as standard care (*n* = 16) and early intervention (*n* = 17) (Figs. 1 and 2). For nine infants in each group, both cord blood at birth and peripheral blood at discharge were collected (Fig. 2).

Baseline and perinatal characteristics for the standard care and early intervention groups are described in Table 1, and no differences were observed among the groups in terms of maternal and infant characteristics, or incidence of postnatal morbidities during NICU stay. Of note, infants enrolled in the study were discharged around term equivalent age (TEA) with no significant differences between the two groups (Table 1).

**Table 1:** Baseline characteristics of the population: descriptive statistics and comparisons between early intervention and standard care groups

Demographic feature	Standard care (*n* = 16)	Early intervention (*n* = 18)	*p* value
Maternal characteristics			
Maternal age (years), mean ± SD	35.1 ± 6.1	33.1 ± 4.8	0.293^^^
Socio-economic status, mean ± SD	47.8 ± 15.8	51.3 ± 9.3	0.627*
Maternal smoking before or during pregnancy, *n* (%)	2 (12%)	1 (6%)	0.591°
Maternal alcohol abuse during pregnancy, *n* (%)	0 (0%)	0 (0%)	> 0.999°
Maternal Body Mass Index, mean ± SD	20.9 ± 4.5	21.8 ± 2.1	0.473
Clinical chorioamnionitis, *n* (%)	8 (50%)	5 (28%)	0.291°
Infant characteristics			
Gestational age at birth (weeks), mean ± SD	27.9 ± 1.1	28.1 ± 1.4	0.318*
Birth weight (g), mean ± SD	1089 ± 347	1005 ± 296	0.453^^^
Male, *n* (%)	9 (56%)	9 (50%)	0.744°
Singleton, *n* (%)	6 (38%)	8 (44%)	0.738°
Small for gestational age, *n* (%)	3 (19%)	4 (22%)	> 0.999°
Cesarean section, *n* (%)	14 (88%)	18 (100%)	0.214°
Apgar score at 1′, median (range)	7 (2–8)	6 (4–8)	0.832*
Apgar score at 5′, median (range)	8 (5–9)	8 (6–9)	0.409*
Clinical Risk Index for Babies (CRIB) II score, mean ± SD	8.0 ± 2.5	8.1 ± 2.1	0.903*
Days of hospitalization, mean ± SD	79.4 ± 30.7	83.9 ± 27.6	0.654^^^
Gestational age at discharge (weeks), mean ± SD	39.4 ± 3.7	40.1 ± 3.7	0.567^^^
Days in the incubator, mean ± SD	51.9 ± 21.9	55.7 ± 18.8	0.589^^^
Postnatal morbidities			
Days of invasive mechanical ventilation, mean ± SD	4.2 ± 6.3	6.1 ± 8.8	0.914*
Days of non-invasive ventilation (NCPAP + nHFT), mean ± SD	31.9 ± 20.4	49.1 ± 36.1	0.220*
Sepsis, *n* (%)	5 (31%)	11 (61%)	0.101°
Severe bronchopulmonary dysplasia, *n* (%)	2 (12%)	8 (44%)	0.063°
Germinal matrix hemorrhage - intraventricular hemorrhage (GMH - IVH) 1–2, *n* (%)	2 (12%)	2 (11%)	> 0.999°
Retinopathy of prematurity (ROP) any grade, *n* (%)	2 (12%)	2 (11%)	> 0.999°

Values are shown as count (percentage) for categorical variables and means ± standard deviations or median (range) for continuous variables. *P* values were obtained using *t* test (^^^), Mann-Whitney *U* Test (*), or Fisher’s exact test (°) - For definition of postnatal morbidities refer to [38]. NCPAP: nasal continuous positive airway pressure; nHFT: nasal high flow therapy

In the early intervention group, the massage therapy was started by parents at 31.7 ± 1.8 (mean ± SD) weeks of GA and carried out on average 10.0 ± 2.1 times a week. Visual interaction was proposed from 34.9 ± 0.8 weeks of GA and performed on average 7.1 ± 1.8 times a week.

In addition, 20 cord blood samples from healthy full-term infants (named “full-term”) were collected at birth. Mother and infants’ characteristics of the full-term group are presented in Additional file 2: Table S1.

### L1 promoter is hypomethylated in preterm infants at birth and its methylation level is restored upon early maternal care

**Fig. 3:**
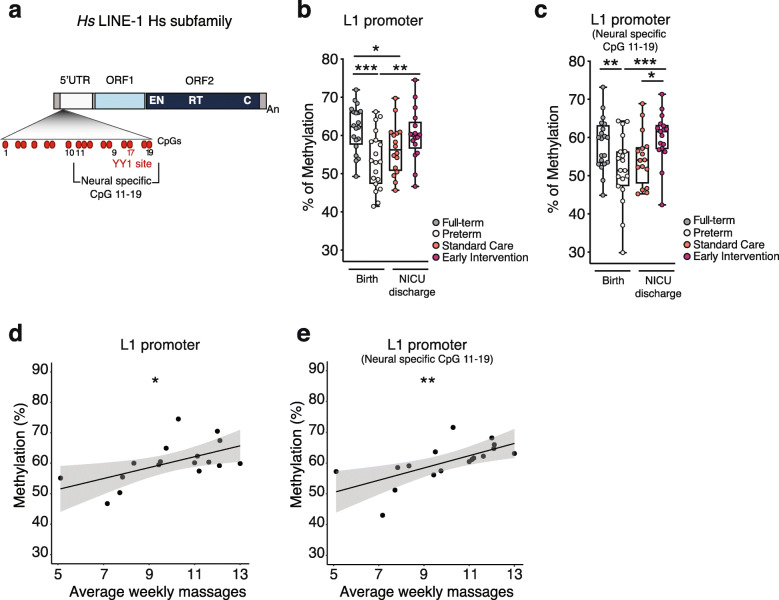
L1 promoter is hypomethylated in preterm neonates and its methylation is restored upon Early Intervention at NICU discharge. **a** Schematic representation of human (*Hs*) LINE-1 (L1): 5′ untranslated region (5′UTR) that contains the internal promoter, open reading frame 1 (ORF1), and open reading frame 2 (ORF2). ORF2 includes endonuclease (EN), reverse transcriptase (RT), and cysteine-rich domains (C); poly (A) tract (An). Within the L1 promoter are highlighted: CpG island (CpG 1–19), the neural-specific CpG (CpG 11–19, as reported in [31]), and YY1-binding site. **b**, **c** Methylation analysis of **b** L1 promoter and **c** L1 promoter neural-specific CpG 11–19 performed on genomic DNA extracted from whole cord blood of full-term (*n* = 20) and preterm neonates (*n* = 19) at birth and from whole peripheral blood of preterm infants at NICU discharge treated with standard care (*n* = 16) or early intervention (*n* = 17). In **b**, ***p < 0.001, full-term vs preterm; *p = 0.015, full-term vs standard care; **p = 0.008, preterm vs early intervention. in **c**, **p = 0.001, full-term vs preterm; ***p < 0.001, preterm vs early Intervention; *p = 0.015, standard care vs early intervention, unpaired two-tailed *t* test. **d**, **e** Scatter plot and linear regression line with 95% confidence band of weekly infant massages vs **d** L1 promoter methylation level (estimate: 1.8, *p* = 0.017) and vs **e** L1 promoter neural-specific CpG 11–19 methylation level (estimate: 2.0, *p* = 0.005) in the early intervention group (*n* = 17)

To assess L1 methylation level, we analyzed a region within the promoter of L1Hs, the evolutionary youngest and most active L1 subfamily in the human genome [50], containing the CpG island already reported to modulate L1 transcription and activity [31]; this CpG island is constituted of 19 CpGs, of which CpG 11–19 are specifically involved in L1 regulation in the brain (Fig. 3a, Neural specific CpG 11–19) [31]; this CpGs subset comprises YY1 transcription factor binding site (CpG 17), required for neural-specific L1 expression [51] (Fig. 3a).

L1 promoter methylation level was analyzed as described in [31] (see the “Methods” section) in whole cord blood of full-term (*n* = 20) and preterm (*n* = 19) infants at birth and in whole peripheral blood of preterm infants at NICU discharge, subjected either to standard care (*n* = 16) or early intervention (*n* = 17) (Figs. 1 and 2, Table 1, Additional file 2: Table S1). We found that L1 promoter methylation was significantly lower in all preterm compared to full-term infants at birth (Fig. 3b and Additional file 1: Fig. S1 a-b) and that at NICU discharge, the early intervention group restored L1 methylation to a level comparable to full-term (Fig. 3b and Additional file 1: Fig. S1 a-d).

Notably, L1 methylation recovery in early intervention group was more specific for the neural region of the promoter (CpG 11–19) (Fig. 3c and Additional file 1: Fig. S1 e) and in particular for CpG 17 corresponding to YY1 binding site (Additional file 1: Fig. S1 f). This is particularly evident in the paired comparison of L1 methylation levels, birth versus NICU discharge; indeed, the subgroup subjected to early intervention displayed increased L1 promoter methylation (Additional file 1: Fig. S1 g-h). We asked whether the different L1 methylation levels could depend on different compositions in whole blood cell types between full-term and preterm infants at birth. Therefore, we analyzed both the proportion and the L1 methylation levels in granulocytes and lymphocytes, the two most abundant blood cell types (up to 80% of nucleated cells) that are known to differ in proportion in whole blood composition among full-term and preterm infants [52, 53]. We found that L1 methylation levels did not differ among granulocytes and lymphocytes (Additional file 1: Fig. S2 a,b), indicating that cord blood cell composition cannot affect the results.

Noteworthy, L1 promoter methylation level increased proportionally to the maternal care received upon early intervention, quantified as the average number of massages received per week, recorded by parents in a daily diary (Fig. 3d), a trend specific for the neural region of L1 promoter (CpG 11–19) (Fig. 3e) and not for CpG 1–10 (Additional file 1: Fig. S2 c).

### L1 activity is fine-tuned during hippocampus and cortex development in mice

We next analyzed L1 methylation in brain and blood mice tissues to inspect L1 dynamics during mammalian brain development and across tissues.

**Fig. 4:**
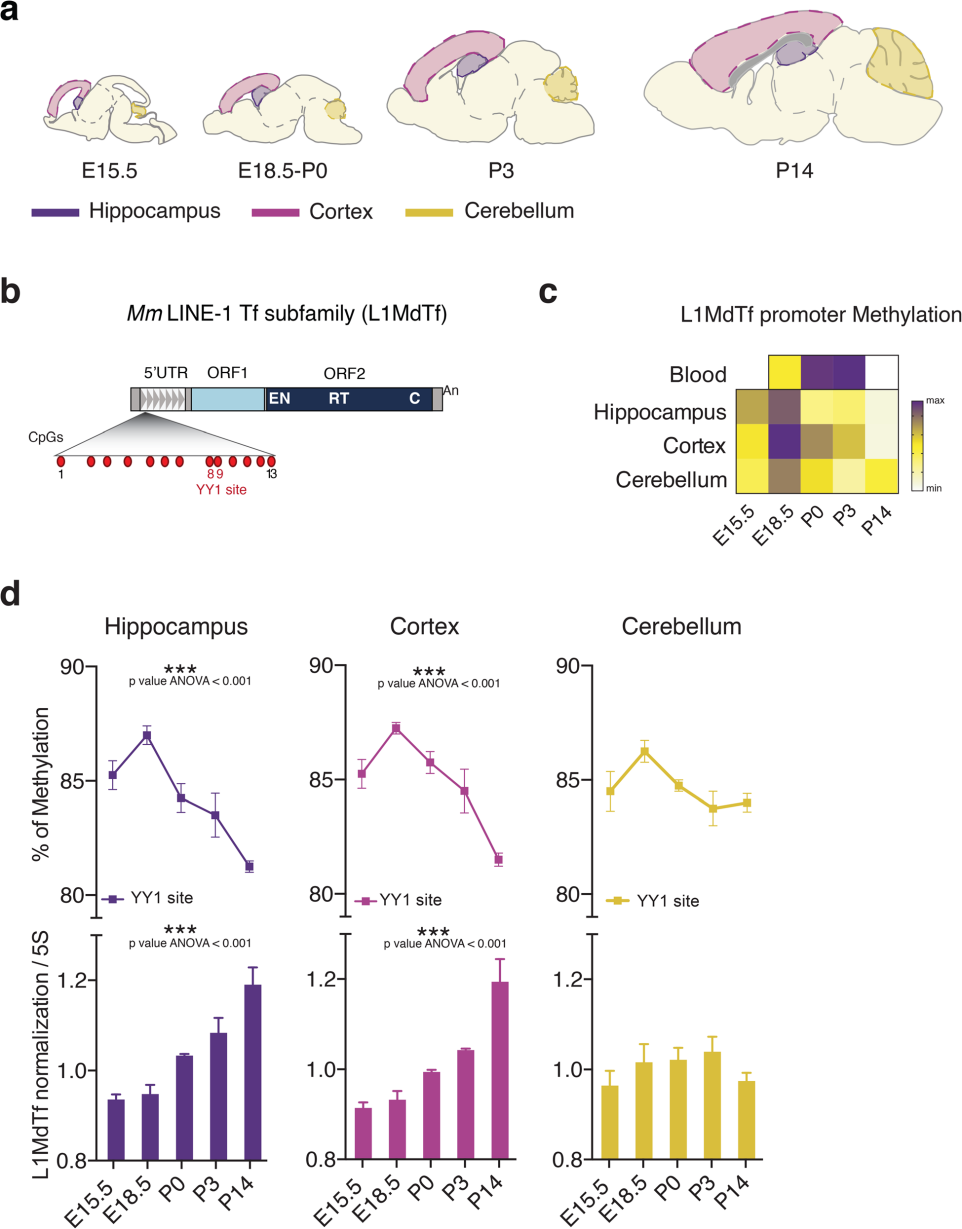
L1 promoter methylation levels and CNVs are dynamic in mouse hippocampus and cortex development. **a** Schematic drawing representing sagittal sections of mouse brain at different stages of embryonic-perinatal (E15.5, E18.5, P0) and postnatal (P3, P14) development. The micro-dissected regions (hippocampus, cerebral cortex, and cerebellum) are highlighted in different colors. **b** Schematic representation of mouse (*Mm*) LINE-1 (L1) Tf subfamily (L1MdTf): 5′ untranslated region (5’UTR), monomeric repeats (grey triangles), open reading frame 1 (ORF1), and open reading frame 2 (ORF2) (ORF2 includes endonuclease (EN), reverse transcriptase (RT), and cysteine-rich domains (C)), poly (A) tract (An). Within the L1 5′UTR monomer are highlighted: CpG island (CpG 1–13) and YY1 binding site (red), as reported in [37]. **c** Heat-map showing methylation levels of L1MdTf promoter in blood, hippocampus, cortex, and cerebellum at different stages of embryonic (E15.5, E18.5) and postnatal development (P0, P3, P14). For each organ and developmental stage, samples from 4 different embryos/mice were analyzed. For blood P0 (*n* = 3) (see the “Methods” section). On an average, 80,000 reads were analyzed for each sample. **d** Matched L1MdTf methylation and L1 CNV analysis in hippocampus, cortex, and cerebellum at different stages of embryonic (E15.5, E18.5) and postnatal development (P0, P3, P14). Upper panels: methylation analysis of L1MdTf promoter at YY1 binding site in the hippocampus, cortex, and cerebellum at different stages of embryonic (E15.5, E18.5) and postnatal development (P0, P3, P14). On an average, 80,000 reads were analyzed for each sample. Hippocampus: E15.5 vs P14, *p* = 0.003; E18.5 vs P0, *p* = 0.047; E18.5 vs P3, *p* = 0.009; E18.5 vs P14, *p* = 0.000; P0 vs P14; *p* = 0.027. Cortex: E15.5 vs P14, *p* = 0.003; E18.5 vs P3, *p* = 0.031; E18.5 vs P14, *p* = 0.000; P0 vs P14, *p* = 0.001; P3 vs P14, *p* = 0.017, ANOVA with Tukey’s post hoc test. Data are represented as the mean percentage of methylation ± S.E.M. Lower panels: L1 CNV assay performed on mouse hippocampus, cortex, and cerebellum at different stages of embryonic (E15.5, E18.5) and postnatal development (P0, P3, P14), obtained from the same 4 mice above. mL1-ORF2 was normalized on m5S. Hippocampus: E15.5 vs P3, *p* = 0.006; E15.5 vs P14, *p* = 0.000; E18.5 vs P3, *p* = 0.012; E18.5 vs P14, *p* = 0.000; P0 vs P14, *p* = 0.004. Cortex: E15.5 vs P3, *p* = 0.018; E15.5 vs P14, *p* = 0.000; E18.5 vs P3, *p* = 0.047; E18.5 vs P14, *p* = 0.000; P0 vs P14, *p* = 0.000; P3 vs P14, *p* = 0.005, ANOVA with Tukey’s post hoc test. Data are represented as mean ± S.E.M

We performed L1 methylation analysis as reported in [37] (see the “Methods” section) on the L1MdTf family that, as human L1Hs, is the most active and evolutionary young L1 subfamily in mice [54]; L1MdTf 5′UTR is constituted by several monomers, each containing a CpG island of 13 CpGs with a YY1 binding site corresponding to the CpG 8 and 9 [55] (Fig. 4b).

We dissected presumptive somatosensory cortex, hippocampus, and cerebellum from the mouse brain at prenatal (E15.5 and E18.5), early postnatal (P0, P3), and late developmental stages (P14) (Fig. 4a); from all these data points, we collected also matched blood samples (with the exception of the E15.5, for material limitation). We observed waves of L1 promoter methylation during development both in blood and in brain tissues (Fig. 4c). In particular, in blood, we found that L1 methylation dynamic is similar to that observed in humans, being the L1 promoter more methylated in early postnatal stages (P0–P3 in mice and full-term at birth in humans) compared with the prenatal window (E18.5 in mice and preterm at birth in humans). These data suggest a concordance between preclinical and clinical data. L1 methylation level was further reduced later postnatally (P14). In brain, we observed similar L1 methylation and demethylation dynamics, although the peak occurred earlier at E18.5 prenatal stage, a developmental window corresponding to that of prematurely born infants (25–29 GA, see the “Discussion” section) [56]. We further analyzed the methylation level at YY1 binding site, already demonstrated to specifically regulate L1MdTf activity in mouse hippocampus [37]; this region displayed a remarkable trend specifically along hippocampus and cerebral cortex developmental trajectories, showing a wave of methylation at E18.5 followed by progressive demethylation soon after birth and postnatally (Fig. 4d upper panels).

To assess whether these methylation dynamics were specific for L1 promoter, we analyzed IAPLTR1a (TEs belonging to ERV superfamily) methylation as reported in [37] (see the “Methods” section) and found no changes both in hippocampus and cortex (Additional file 1: Fig. S3a). Conversely, IAPLTR1a methylation level decreases postnatally in the cerebellum (Additional file 1: Fig. S3 a, right panel), being L1 methylation unchanged. These data suggest that different regions of the brain display specific methylation dynamics of different TEs during development.

We next investigated whether the reduction in L1 methylation observed postnatally in mouse hippocampus and cortex could correspond to an increased L1 CNVs, as L1 are reported to retrotranspose in the brain [57]. We measured L1 CNVs in genomic DNA as reported in [31] (Additional file 1: Figure S3b), treating the gDNA with Exonuclease I in order to avoid the amplification of L1 cDNA intermediates [37] (Additional file 1: Fig. S3 c). Interestingly, we observed a statistically significant increase in L1 CNVs in the hippocampus and cerebral cortex postnatally (P0, P3, P14) that corresponds to a decrease in L1 promoter methylation (Fig. 4d). Cerebellum samples did not show any change in L1 CNVs (Fig. 4d lower panels).

Overall, these results suggest that L1 activity is fine-tuned and specifically regulated during hippocampus and cerebral cortical development, and identify E18.5 as a sensitive and “vulnerable” developmental stage for the epigenetic setting of L1 methylation and activity regulation in these brain regions.

### Early maternal care enhances neurodevelopmental outcomes in preterm infants

Based on the hypothesis that L1 modulation in the perinatal period may represent one of the molecular mechanisms involved in the modulation of the infant’s long-term neurodevelopment, we report the results of the post hoc analysis on neurodevelopmental assessment performed with the Griffiths Scales at 12 months corrected age and 36 months chronological age in this subgroup of the larger RCT cohort [43, 44] (Fig. 1). At 12 months corrected age, one infant in the standard care group was lost to follow-up, due to severe illness that required prolonged hospitalization after NICU discharge, and one infant in the early intervention group was excluded from the follow-up evaluation as non-cystic white matter damage was observed at brain MRI performed at 40+ 0 GA (Fig. 2). In addition, 3 infants in the early intervention group did not undergo the 36-month assessment as they were lost at follow-up (Fig. 2).

On average, all the preterm infants showed developmental scores within the normal range; however, statistically significant differences were observed between the 2 groups with the early intervention group showing higher scores (Table 2), both at 12 and 36 months, in the general quotient and in 4 out of 5 subscales: personal-social (that measures proficiency in the activities of daily living, level of independence and interaction with other children), hearing and language (that assesses hearing, expressive language, and receptive language), eye and hand coordination (that tests fine motor skills, manual dexterity, and visual monitoring skills), and performance (that evaluates the ability to reason through tasks including speed of working and precision) (Table 2). No differences were observed in the locomotor subscale that measures gross motor skills, including the ability to balance, coordinate, and control movements (Table 2).

**Table 2:** Neurodevelopmental outcome at 12 months corrected age and 36 months chronological age

	Standard care	Early intervention	*p* value
12 months follow-up	*n* = 15	*n* = 17	
General quotient, mean ± SD	90.5 ± 3.3	93.9 ± 4.4	0.017^^^
Locomotor, mean ± SD	96.3 ± 5.7	94.9 ± 9.8	0.894*
Personal-social, mean ± SD	87.5 ± 4.6	93.9 ± 5.1	0.001^^^
Hearing and language, mean ± SD	91.0 ± 4.2	94.8 ± 3.8	0.024*
Eye and hand coordination, mean ± SD	89.5 ± 6.2	94.7 ± 4.8	0.014^^^
Performance, mean ± SD	91.1 ± 3.9	94.8 ± 5.6	0.026*
36 months follow-up	*n* = 15	*n* = 14	
General quotient, mean ± SD	86.5 ± 4.7	90.7 ± 4.8	0.026^^^
Locomotor, mean ± SD	91.3 ± 4.0	91.9 ± 5.5	0.744^^^
Personal-social, mean ± SD	85.0 ± 5.3	89.8 ± 4.1	0.009*
Hearing and language, mean ± SD	86.1 ± 7.4	92.3 ± 5.2	0.014^^^
Eye and hand coordination, mean ± SD	86.8 ± 5.9	92.4 ± 4.7	0.009^^^
Foundation of learning^a^, mean ± SD	89.0 ± 5.3	93.7 ± 3.4	0.008^^^

Means ± standard deviations are shown. *P* values were obtained using *t* test (^^^) or Mann-Whitney *U* test (*). ^a^This subscale corresponds to “Performance” in GMDS-R

Overall, these results show that in this cohort the early intervention ameliorates the neurodevelopmental outcomes of premature infants already at the short-term (12 months corrected age) and, importantly, this trend is maintained later in childhood (36 months), suggesting a positive long-lasting modulation of their neurodevelopment. These findings were confirmed by the post hoc analysis performed on the whole cohort enrolled in the RCT (Additional file 2: Table S2).

## Discussion

Here we report that preterm infants born before 30 weeks of GA display L1 promoter hypomethylation at birth compared to healthy full-term newborns; this result is in line with previous observations [58], and we further demonstrated that L1 methylation status in preterm infants can be restored by the beneficial effect of early maternal care and positive multisensory experiences. The L1 methylation results could be affected by clinical and/or other confounding factors; however, the potential of such confounders might have been limited by the fact that the cohort represents a homogeneous population of a larger cohort that derived from a randomized trial (see the “Methods” section). In addition, we documented that the early intervention strategy positively modulates infants’ neurodevelopment.

The early intervention we have adopted lays its theoretical basis on environmental enrichment that refers to positive active experiences enhancing a functional and structural brain reorganization in infancy [59, 60]; here we have combined both protective effect of an empowered maternal care with a positive multisensory experience during a critical period for brain development [20, 61].

Maternal separation and excessive sensory exposure, related to NICU environment, represent early adverse life events that can affect the epigenetic regulation and impact gene expression in prematurely born infants [62]. This is, to our knowledge, the first study that documents an epigenetic modulation, specifically on L1 repetitive sequences, induced by maternal care and multisensory stimulation in humans.

It is very well demonstrated that de novo L1 insertions are a fine-tuned, developmentally regulated phenomenon that contributes to genomic somatic mosaicism of the brain [63]. However, L1 deregulated activity could have detrimental effects as reported for several mental disorders including schizophrenia (SCZ), autism spectrum disorders (ASD), and major depression [36], which often occur in adulthood of individuals born preterm [64]. In this context, we dissected L1 dynamics in the developing mouse brain, by sampling distinct regions (cerebral cortex, hippocampus, and cerebellum), and we detected increased L1 CNVs at birth (P0 in the mouse) that paralleled the progressive reduction in L1 promoter methylation (starting from E18.5) in specific brain areas, such as the hippocampus and cerebral cortex. Quantifying copy number variation for transposable elements is challenging due to the low frequency of de novo insertions in respect to the baseline of the preexisting copies. While early developmental insertions are likely to be clonally expanded resulting in several cells owning the insertions, very rare somatic insertions can be distinguished only with accurate tools. Although methodologies with SYBR Green have been extensively adopted [32, 33], the use of Taqman probes [31] and the advent of droplet digital PCR (dPCR) [37] have improved the sensitivity of LINE1 CNVs measurement. In particular, dPCR represents the most sensitive tool to detect very rare somatic CNVs, further, as it can distinguish single nucleotide polymorphisms and so specific L1 subfamilies, it is suitable also for diagnostic purposes [65].

In the current work, we have identified a susceptible prenatal time window in mice, peaking around E18.5 (2–3 days before natural birth) in which L1 epigenetic regulation is set (Fig. 4). Interestingly, the hippocampus and the interconnected cerebral cortex, as well thalamus, are known to be affected by preterm birth in humans to an extent proportional to the degree of prematurity [66]. Therefore, it is tempting to speculate that similarly to mice, L1 dynamics could occur also in human brain development and that premature extrauterine life exposure can have an impact on these fine-tuned phenomena. The fundamental differences in pregnancy duration, as well as the human-specific characteristics of neocortical development, such as cortical expansion, protracted neuron maturation (i.e., neoteny) [67], and genetics, pose a significant challenge in directly comparing neuronal development between species. Recently, comparative transcriptomic studies—at single cell resolution—of the cortex and hippocampus [68–70] indicate species-specific differences in the cellular makeup of human brain [71, 72], while identifying divergent molecular and functional features of conserved neuronal classes [73]. However, the general principles underlying cortical development and basic cortical architecture appear to be conserved across mammals, including humans [74]. Common developmental milestones have been described in particular during the stages prior to birth in both the human and mouse cortex and hippocampus that might determine the correct assembly and functioning of neural circuits in both species, such as, for example, local and commissural connectivity dynamics. Indeed, in utero functional MRI studies have shown that functional connectivity is established in human already before birth (GA 21–38), and particularly synchronicity increases at the transition from the second to the third trimester, with the peak around GA 26–29, mainly due to short-range intrahemispheric and interhemispheric/commissural connections [75]. Similarly, in mouse cortex around E17.5 commissural axons from neurons of the cingulate cortex begin the process of midline crossing acting as pioneers for neocortical callosal neurons, which begin to cross only 1 day later (E18.5), eventually establishing the first interhemispheric connections [76, 77]. Furthermore, GABAergic synaptic responses in rodents can be already recorded at late embryonic stages (i.e., E18) in the mouse neocortex [78] and hippocampus [79], supporting the notion that also short-range functional networks, albeit still immature, have been established by then [80].

Thus, even without directly aligning the human and mouse neurodevelopmental trajectories, a defined prenatal time window in each species (GA 26–29 for human and E17.5–E18.5 for mouse) that hosts critical events shaping brain architecture and networks maturation has been identified [75]. Alterations of these connectivity patterns and/or of the underlying epigenetic settings (as potentially induced by premature birth) may lead to long-lasting neuronal deficits. However, further studies are required to precisely dissect the mechanisms that subtend neurological alterations in prematurity and their consequences both at molecular level on L1 activity and to investigate potential species-specific mechanisms at play in particular on human brain development.

Importantly, we further demonstrated that the early intervention program, compared to standard care, enhances neurodevelopment up to 36 months of age, which is considered a crucial milestone in the preterms’ follow-up assessment to detect neurodevelopmental disabilities [4]. However, follow-up at school age is still ongoing to ultimately confirm these beneficial effects of early intervention.

## Conclusions

In conclusion, we are providing evidence that L1 methylation is fine-tuned at prenatal stages in humans and in mouse brain; we can speculate that this developmental window is therefore extremely sensible in preterm infants to the effects of maternal care on the regulation of the L1 epigenetic setting.

This work shed light on a possible molecular function for L1 activity in shaping the developing brain connections with potential impact on infants’ neurodevelopment. Besides this mechanistic role of L1, this study supports a so far unexplored translational approach to prematurity-related disorders: L1 methylation analysis on an extended cohort of preterm newborns would be required to define its predictive value as a molecular proxy for neurodevelopmental outcomes.

## Supplementary Information

**Additional file 1: Figure S1.** Early Intervention affects methylation of YY1 binding site in L1 promoter. **Figure S2.** Granulocytes and lymphocytes isolated from cord blood of full-term and preterm display similar L1 methylation. **Figure S3.** IAP methylation levels do not change in hippocampus and cortex development while reduce in cerebellum development.

**Additional file 2: Table S1.** Baseline characteristics of the population: descriptive statistics and comparisons between Full-term and Preterm (both Early Intervention and Standard Care) groups. **Table S2.** Neurodevelopmental outcome at 12 months corrected age and 36 months chronological age of the overall population included in the RCT. **Table S3.** List of the primer sequences used in L1MdTf and IAPLTR1a NGS methylation analysis in mice.

## Data Availability

All data needed to evaluate the conclusions are present in the paper and/or the supplementary materials. Additional data related to this paper may be available upon request. The data from bisulfite sequencing has been submitted in NCBI GEO (GSE136844).

## Abbreviations

L1: LINE-1, long interspersed nuclear element 1
NICU: Neonatal intensive care unit
GA: Gestational age
MRI: Magnetic resonance imaging
RTase: Reverse transcriptase
CNVs: Copy number variations
RCT: Randomized controlled trial
TEA: Term equivalent age
GMDS: Griffiths Mental Development Scales

## References

[1] Blencowe H, Cousens S, Chou D, Oestergaard M, Say L, Moller AB, Kinney M, Lawn J, Born Too Soon Preterm Birth Action G (2013). Born too soon: the global epidemiology of 15 million preterm births. Reprod Health.

[2] Purisch SE, Gyamfi-Bannerman C (2017). Epidemiology of preterm birth. Semin Perinatol.

[3] Moster D, Lie RT, Markestad T (2008). Long-term medical and social consequences of preterm birth. N Engl J Med.

[4] Moore T, Hennessy EM, Myles J, Johnson SJ, Draper ES, Costeloe KL, Marlow N (2012). Neurological and developmental outcome in extremely preterm children born in England in 1995 and 2006: the EPICure studies. BMJ.

[5] Aarnoudse-Moens CS, Weisglas-Kuperus N, van Goudoever JB, Oosterlaan J (2009). Meta-analysis of neurobehavioral outcomes in very preterm and/or very low birth weight children. Pediatrics.

[6] Montagna A, Nosarti C (2016). Socio-emotional development following very preterm birth: pathways to psychopathology. Front Psychol.

[7] Johnson S, Marlow N (2017). Early and long-term outcome of infants born extremely preterm. Arch Dis Child.

[8] Pineda R, Guth R, Herring A, Reynolds L, Oberle S, Smith J (2017). Enhancing sensory experiences for very preterm infants in the NICU: an integrative review. J Perinatol.

[9] Smyser TA, Smyser CD, Rogers CE, Gillespie SK, Inder TE, Neil JJ (2016). Cortical gray and adjacent white matter demonstrate synchronous maturation in very preterm infants. Cereb Cortex.

[10] Fu M, Zuo Y (2011). Experience-dependent structural plasticity in the cortex. Trends Neurosci.

[11] Mooney-Leber SM, Brummelte S (2017). Neonatal pain and reduced maternal care: early-life stressors interacting to impact brain and behavioral development. Neuroscience.

[12] Volpe JJ (2019). Dysmaturation of premature brain: importance, cellular mechanisms, and potential interventions. Pediatr Neurol.

[13] Corcoles-Parada M, Gimenez-Mateo R, Serrano-Del-Pueblo V, Lopez L, Perez-Hernandez E, Mansilla F, Martinez A, Onsurbe I, San Roman P, Ubero-Martinez M (2019). Born too early and too small: higher order cognitive function and brain at risk at ages 8-16. Front Psychol.

[14] Fenoglio A, Georgieff MK, Elison JT (2017). Social brain circuitry and social cognition in infants born preterm. J Neurodev Disord.

[15] Lodato S, Arlotta P (2015). Generating neuronal diversity in the mammalian cerebral cortex. Annu Rev Cell Dev Biol.

[16] Mancinelli S, Lodato S (2018). Decoding neuronal diversity in the developing cerebral cortex: from single cells to functional networks. Curr Opin Neurobiol.

[17] Haebich KM, Willmott C, Scratch SE, Pascoe L, Lee KJ, Spencer-Smith MM, Cheong JLY, Inder TE, Doyle LW, Thompson DK, et al. Neonatal brain abnormalities and brain volumes associated with goal setting outcomes in very preterm 13-year-olds. Brain Imaging Behav. 2020;14:1062–73. 10.1007/s11682-019-00039-1 30684152

[18] Ball G, Boardman JP, Aljabar P, Pandit A, Arichi T, Merchant N, Rueckert D, Edwards AD, Counsell SJ (2013). The influence of preterm birth on the developing thalamocortical connectome. Cortex.

[19] Youssef M, Atsak P, Cardenas J, Kosmidis S, Leonardo ED, Dranovsky A (2019). Early life stress delays hippocampal development and diminishes the adult stem cell pool in mice. Sci Rep.

[20] Milgrom J, Newnham C, Anderson PJ, Doyle LW, Gemmill AW, Lee K, Hunt RW, Bear M, Inder T (2010). Early sensitivity training for parents of preterm infants: impact on the developing brain. Pediatr Res.

[21] Als H, Duffy FH, McAnulty GB, Rivkin MJ, Vajapeyam S, Mulkern RV, Warfield SK, Huppi PS, Butler SC, Conneman N (2004). Early experience alters brain function and structure. Pediatrics.

[22] Vinall J, Miller SP, Synnes AR, Grunau RE (2013). Parent behaviors moderate the relationship between neonatal pain and internalizing behaviors at 18 months corrected age in children born very prematurely. Pain.

[23] Lordier L, Loukas S, Grouiller F, Vollenweider A, Vasung L, Meskaldij DE, Lejeune F, Pittet MP, Borradori-Tolsa C, Lazeyras F (2019). Music processing in preterm and full-term newborns: a psychophysiological interaction (PPI) approach in neonatal fMRI. Neuroimage.

[24] Pineda R, Raney M, Smith J (2019). Supporting and enhancing NICU sensory experiences (SENSE): defining developmentally-appropriate sensory exposures for high-risk infants. Early Hum Dev.

[25] Beck CR, Garcia-Perez JL, Badge RM, Moran JV (2011). LINE-1 elements in structural variation and disease. Annu Rev Genomics Hum Genet.

[26] Faulkner GJ, Garcia-Perez JL (2017). L1 mosaicism in mammals: extent, effects, and evolution. Trends Genet.

[27] Baillie JK, Barnett MW, Upton KR, Gerhardt DJ, Richmond TA, De Sapio F, Brennan PM, Rizzu P, Smith S, Fell M (2011). Somatic retrotransposition alters the genetic landscape of the human brain. Nature.

[28] Perrat PN, DasGupta S, Wang J, Theurkauf W, Weng Z, Rosbash M, Waddell S (2013). Transposition-driven genomic heterogeneity in the Drosophila brain. Science.

[29] Upton KR, Gerhardt DJ, Jesuadian JS, Richardson SR, Sanchez-Luque FJ, Bodea GO, Ewing AD, Salvador-Palomeque C, van der Knaap MS, Brennan PM (2015). Ubiquitous L1 mosaicism in hippocampal neurons. Cell.

[30] Muotri AR, Chu VT, Marchetto MC, Deng W, Moran JV, Gage FH (2005). Somatic mosaicism in neuronal precursor cells mediated by L1 retrotransposition. Nature.

[31] Coufal NG, Garcia-Perez JL, Peng GE, Yeo GW, Mu Y, Lovci MT, Morell M, O'Shea KS, Moran JV, Gage FH (2009). L1 retrotransposition in human neural progenitor cells. Nature.

[32] Muotri AR, Marchetto MC, Coufal NG, Oefner R, Yeo G, Nakashima K, Gage FH (2010). L1 retrotransposition in neurons is modulated by MeCP2. Nature.

[33] Bundo M, Toyoshima M, Okada Y, Akamatsu W, Ueda J, Nemoto-Miyauchi T, Sunaga F, Toritsuka M, Ikawa D, Kakita A (2014). Increased l1 retrotransposition in the neuronal genome in schizophrenia. Neuron.

[34] Shpyleva S, Melnyk S, Pavliv O, Pogribny I, Jill James S (2018). Overexpression of LINE-1 retrotransposons in autism brain. Mol Neurobiol.

[35] Lapp HE, Hunter RG (2019). Early life exposures, neurodevelopmental disorders, and transposable elements. Neurobiol Stress.

[36] Misiak B, Ricceri L, Sasiadek MM (2019). Transposable elements and their epigenetic regulation in mental disorders: current evidence in the field. Front Genet.

[37] Bedrosian TA, Quayle C, Novaresi N, Gage FH (2018). Early life experience drives structural variation of neural genomes in mice. Science.

[38] Fontana C, Menis C, Pesenti N, Passera S, Liotto N, Mosca F, Roggero P, Fumagalli M (2018). Effects of early intervention on feeding behavior in preterm infants: a randomized controlled trial. Early Hum Dev.

[39] Newnham CA, Milgrom J, Skouteris H (2009). Effectiveness of a modified Mother-Infant Transaction Program on outcomes for preterm infants from 3 to 24 months of age. Infant Behav Dev.

[40] Fontana C, De Carli A, Ricci D, Dessimone F, Passera S, Pesenti N, Bonzini M, Bassi L, Squarcina L, Cinnante C (2020). Effects of early intervention on visual function in preterm infants: a randomized controlled trial. Front Pediatr.

[41] Stelzer GT, Shults KE, Loken MR (1993). CD45 gating for routine flow cytometric analysis of human bone marrow specimens. Ann N Y Acad Sci.

[42] Lacombe F, Durrieu F, Briais A, Dumain P, Belloc F, Bascans E, Reiffers J, Boisseau MR, Bernard P (1997). Flow cytometry CD45 gating for immunophenotyping of acute myeloid leukemia. Leukemia.

[43] Griffiths R (1970). The abilities of young children: a comprehensive system of mental measurement for the first eight years of life.

[44] Stroud LFC, Green E, Bloomfield S, Cronje J, Hurter K, Lane H, Marais R, Marx C, McAlinden P, O’Connell R, Paradice R, Venter D (2016). Griffiths Scales of Child Development.

[45] Li Y, Tollefsbol TO (2011). DNA methylation detection: bisulfite genomic sequencing analysis. Methods Mol Biol.

[46] Kumaki Y, Oda M, Okano M (2008). QUMA: quantification tool for methylation analysis. Nucleic Acids Res.

[47] Bolger AM, Lohse M, Usadel B (2014). Trimmomatic: a flexible trimmer for Illumina sequence data. Bioinformatics.

[48] Krueger F, Andrews SR (2011). Bismark: a flexible aligner and methylation caller for Bisulfite-Seq applications. Bioinformatics.

[49] Akalin A, Kormaksson M, Li S, Garrett-Bakelman FE, Figueroa ME, Melnick A, Mason CE (2012). methylKit: a comprehensive R package for the analysis of genome-wide DNA methylation profiles. Genome Biol.

[50] Beck CR, Collier P, Macfarlane C, Malig M, Kidd JM, Eichler EE, Badge RM, Moran JV (2010). LINE-1 retrotransposition activity in human genomes. Cell.

[51] Sanchez-Luque FJ, Kempen MHC, Gerdes P, Vargas-Landin DB, Richardson SR, Troskie RL, Jesuadian JS, Cheetham SW, Carreira PE, Salvador-Palomeque C, et al. LINE-1 evasion of epigenetic repression in humans. Mol Cell. 2019;75(3):590–604.e12 10.1016/j.molcel.2019.05.024 31230816

[52] Correa-Rocha R, Perez A, Lorente R, Ferrando-Martinez S, Leal M, Gurbindo D, Munoz-Fernandez MA (2012). Preterm neonates show marked leukopenia and lymphopenia that are associated with increased regulatory T-cell values and diminished IL-7. Pediatr Res.

[53] Milcic TL (2009). The complete blood count. Neonatal Netw.

[54] Sookdeo A, Hepp CM, McClure MA, Boissinot S (2013). Revisiting the evolution of mouse LINE-1 in the genomic era. Mob DNA.

[55] Lee SH, Cho SY, Shannon MF, Fan J, Rangasamy D (2010). The impact of CpG island on defining transcriptional activation of the mouse L1 retrotransposable elements. PLoS One.

[56] Carson R, Monaghan-Nichols AP, DeFranco DB, Rudine AC (2016). Effects of antenatal glucocorticoids on the developing brain. Steroids.

[57] Richardson SR, Morell S, Faulkner GJ (2014). L1 retrotransposons and somatic mosaicism in the brain. Annu Rev Genet.

[58] Michels KB, Harris HR, Barault L (2011). Birthweight, maternal weight trajectories and global DNA methylation of LINE-1 repetitive elements. PLoS One.

[59] Baroncelli L, Braschi C, Spolidoro M, Begenisic T, Sale A, Maffei L (2010). Nurturing brain plasticity: impact of environmental enrichment. Cell Death Differ.

[60] Purpura G, Cioni G, Tinelli F (2017). Multisensory-based rehabilitation approach: translational insights from animal models to early intervention. Front Neurosci.

[61] Guzzetta A, Baldini S, Bancale A, Baroncelli L, Ciucci F, Ghirri P, Putignano E, Sale A, Viegi A, Berardi N (2009). Massage accelerates brain development and the maturation of visual function. J Neurosci.

[62] Fumagalli M, Provenzi L, De Carli P, Dessimone F, Sirgiovanni I, Giorda R, Cinnante C, Squarcina L, Pozzoli U, Triulzi F (2018). From early stress to 12-month development in very preterm infants: preliminary findings on epigenetic mechanisms and brain growth. PLoS One.

[63] Bodea GO, McKelvey EGZ, Faulkner GJ. Retrotransposon-induced mosaicism in the neural genome. Open Biol. 2018;8(7):180074. 10.1098/rsob.180074 PMC6070720 30021882

[64] Mathewson KJ, Chow CH, Dobson KG, Pope EI, Schmidt LA, Van Lieshout RJ (2017). Mental health of extremely low birth weight survivors: a systematic review and meta-analysis. Psychol Bull.

[65] Newkirk SJ, Kong L, Jones MM, Habben CE, Dilts VL, Ye P, An W (2020). Subfamily-specific quantification of endogenous mouse L1 retrotransposons by droplet digital PCR. Anal Biochem.

[66] Ball G, Boardman JP, Rueckert D, Aljabar P, Arichi T, Merchant N, Gousias IS, Edwards AD, Counsell SJ (2012). The effect of preterm birth on thalamic and cortical development. Cereb Cortex.

[67] Petanjek Z, Judas M, Simic G, Rasin MR, Uylings HB, Rakic P, Kostovic I (2011). Extraordinary neoteny of synaptic spines in the human prefrontal cortex. Proc Natl Acad Sci U S A.

[68] Fan X, Fu Y, Zhou X, Sun L, Yang M, Wang M, Chen R, Wu Q, Yong J, Dong J (2020). Single-cell transcriptome analysis reveals cell lineage specification in temporal-spatial patterns in human cortical development. Sci Adv.

[69] Zhong S, Ding W, Sun L, Lu Y, Dong H, Fan X, Liu Z, Chen R, Zhang S, Ma Q (2020). Decoding the development of the human hippocampus. Nature.

[70] Semple BD, Blomgren K, Gimlin K, Ferriero DM, Noble-Haeusslein LJ (2013). Brain development in rodents and humans: identifying benchmarks of maturation and vulnerability to injury across species. Prog Neurobiol.

[71] Boldog E, Bakken TE, Hodge RD, Novotny M, Aevermann BD, Baka J, Borde S, Close JL, Diez-Fuertes F, Ding SL (2018). Transcriptomic and morphophysiological evidence for a specialized human cortical GABAergic cell type. Nat Neurosci.

[72] Molnar Z, Clowry GJ, Sestan N, Alzu'bi A, Bakken T, Hevner RF, Huppi PS, Kostovic I, Rakic P, Anton ES (2019). New insights into the development of the human cerebral cortex. J Anat.

[73] Hodge RD, Bakken TE, Miller JA, Smith KA, Barkan ER, Graybuck LT, Close JL, Long B, Johansen N, Penn O (2019). Conserved cell types with divergent features in human versus mouse cortex. Nature.

[74] Defelipe J (2011). The evolution of the brain, the human nature of cortical circuits, and intellectual creativity. Front Neuroanat.

[75] Jakab A, Schwartz E, Kasprian G, Gruber GM, Prayer D, Schopf V, Langs G (2014). Fetal functional imaging portrays heterogeneous development of emerging human brain networks. Front Hum Neurosci.

[76] Lindwall C, Fothergill T, Richards LJ (2007). Commissure formation in the mammalian forebrain. Curr Opin Neurobiol.

[77] Fame RM, MacDonald JL, Macklis JD (2011). Development, specification, and diversity of callosal projection neurons. Trends Neurosci.

[78] Verhage M, Maia AS, Plomp JJ, Brussaard AB, Heeroma JH, Vermeer H, Toonen RF, Hammer RE, van den Berg TK, Missler M (2000). Synaptic assembly of the brain in the absence of neurotransmitter secretion. Science.

[79] Hennou S, Khalilov I, Diabira D, Ben-Ari Y, Gozlan H (2002). Early sequential formation of functional GABA(A) and glutamatergic synapses on CA1 interneurons of the rat foetal hippocampus. Eur J Neurosci.

[80] Le Magueresse C, Monyer H (2013). GABAergic interneurons shape the functional maturation of the cortex. Neuron.

