# Supplementary material for: Early maternal care restores LINE-1 methylation and enhances neurodevelopment in preterm infants
Source: BMC Med. 2021 Feb 5;19:42. doi: 10.1186/s12916-020-01896-0 (PMC7863536; doi:10.1186/s12916-020-01896-0)
Supplement: Supplementary file 1 — Additional file 1: Figure S1. Early Intervention affects methylation of YY1 binding site in L1 promoter. Figure S2. Granulocytes and lymphocytes isolated from cord blood of full-term and preterm display similar L1 methylation. Figure S3. IAP methylation levels do not change in hippocampus and cortex development while reduce in cerebellum development. [file 12916_2020_1896_MOESM1_ESM.doc]

**Early maternal care restores LINE-1 methylation and enhances neurodevelopment in preterm infants**

Camilla Fontana ^1^†, Federica Marasca ^2^†, Livia Provitera ^3^, Sara Mancinelli ^4,5^, Nicola Pesenti ^3,6^, Shruti Sinha ^2^, Sofia Passera ^3^, Sergio Abrignani ^1,2^, Fabio Mosca ^1,3^, Simona Lodato ^4,5^, Beatrice Bodega ^2^‡*, Monica Fumagalli ^1,3^‡*.

^1^ University of Milan, Department of Clinical Sciences and Community Health, Milan, Italy.

^2^ Istituto Nazionale di Genetica Molecolare “Enrica e Romeo Invernizzi” (INGM), Milan, Italy.

^3^ Fondazione IRCCS Ca’ Granda Ospedale Maggiore Policlinico, NICU, Milan, Italy.

^4^ IRCCS Humanitas Clinical and Research Center, Rozzano - Milan, Italy.

^5^ Humanitas University, Department of Biomedical Sciences, Pieve Emanuele-Milan, Italy.

^6^ Department of Statistics and Quantitative Methods, Division of Biostatistics, Epidemiology and Public Health, University of Milano-Bicocca, Milan, Italy.

† Contributed equally as first author to this work

‡ Contributed equally as last author to this work.

* Correspondence should be addressed to: [monica.fumagalli@unimi.it](mailto:monica.fumagalli@unimi.it) and [bodega@ingm.org](mailto:bodega@ingm.orhg)

**Supplementary Figures and Legends**

**Additional File 1: Figure S1-S3.**

**Figure S1. Early iIntervention affects methylation of YY1 binding site in L1 promoter.**

**Figure S2. Granulocytes and lymphocytes isolated from cord blood of full-term and preterm display similar L1 methylation.**

**Figure S3. IAP methylation levels do not change in hippocampus and cortex development while reduce in cerebellum development.**

**
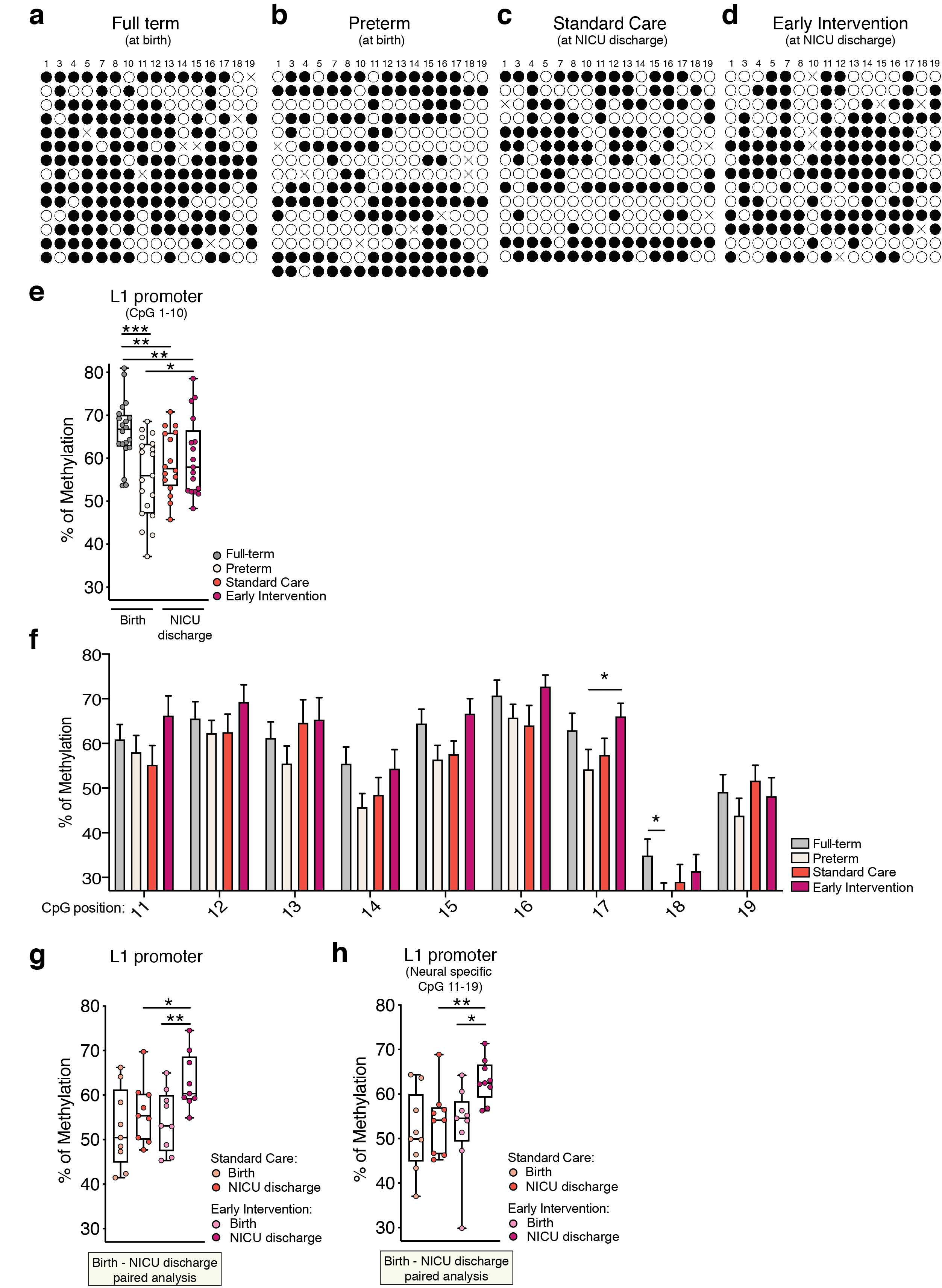
**

**Figure S1. Early Intervention affects methylation of YY1 binding site in L1 promoter**

**a-d** Methylation analysis of L1 promoter in whole cord blood of **a** full-term and **b** preterm at birth and whole peripheral blood of preterm infants at NICU discharge treated with **c** standard care or **d** early intervention. Lollipop diagrams display sequences obtained from a representative sample for each group (black, methylated CpG; white, unmethylated CpG; x, absent CpG). Each row represents an individual Sanger sequence. Numbers at the top correspond to the CpG sites shown in Figure 3a (see Methods). **e** CpG 1-10 methylation analysis in whole cord blood of full-term and preterm neonates at birth and in whole peripheral blood of preterm infants at NICU discharge treated with standard care or early intervention. *** p < 0.001, full-term vs preterm, ** p = 0.001, full-term vs standard Care; ** p = 0.006, full-term vs early intervention; * p = 0.039, preterm vs early intervention, unpaired two-tailed t test. **f** CpG 11-19 methylation analysis at the single CpG level. CpG 17, * p = 0.027, preterm vs early intervention; CpG 18, * p = 0.047, full – term vs preterm, ANOVA with Tukey post hoc test. In **e** and **f**, data are represented as the mean percentage of methylation ± S.E.M. (full-term, n = 20; preterm, n = 19; standard care, n = 16; early intervention, n =17). **g-h** Birth – NICU discharge paired methylation analysis of **g** L1 promoter and **h** L1 promoter neural specific CpG 11-19 analysis in whole cord and whole peripheral blood of preterm infants treated with standard care (n = 9) and with early intervention (n = 9). In **g** **p = 0.007 and in **h** *p = 0.022, birth vs NICU discharge, early intervention, two - tailed paired t test; in **g** *p = 0.041 and in **h** **p = 0.008, early intervention vs standard care, NICU discharge, unpaired two – tailed t test.

**
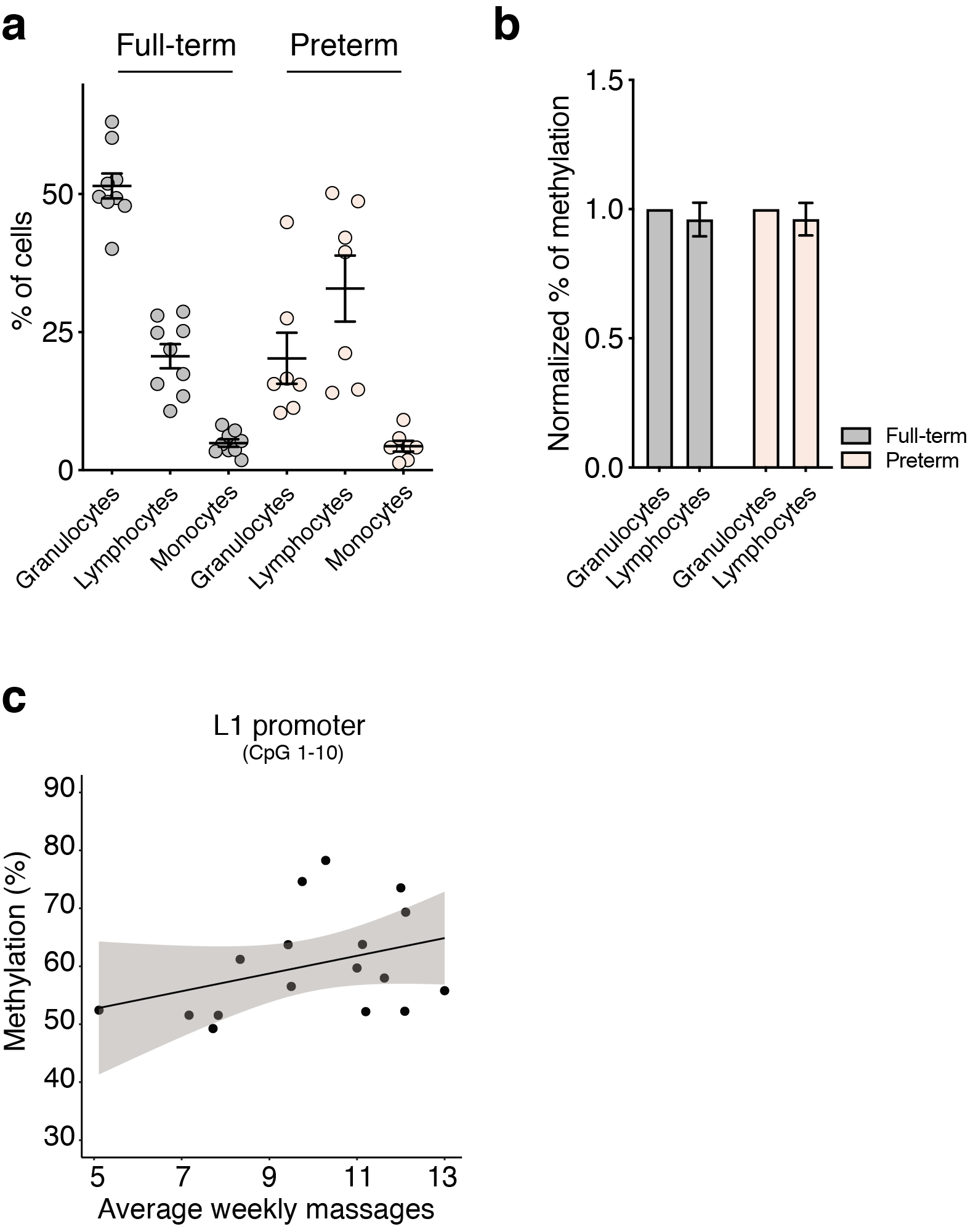
**

**Figure S2. Granulocytes and lymphocytes isolated from cord blood of full-term and preterm display similar L1 methylation**

**a** Phenotypical analysis based on CD45/SSC (see methods) was performed on cord blood of full-term (n = 9) and preterm (n = 7) neonates at birth; granulocytes, lymphocytes and monocytes % of cells are indicated. **b** L1 promoter normalized methylation analysis of granulocytes and lymphocytes isolated from whole cord blood of full-term (n = 6) and preterm (n = 4) neonates at birth. For each individual at least 12 independent randomly selected clones were analyzed. Data are represented as the mean percentage of methylation ± S.E.M. **c** Scatter plot and linear regression line with 95% confidence band of weekly massages vs L1 promoter (CpG 1-10) methylation level in the early intervention group (n = 17).


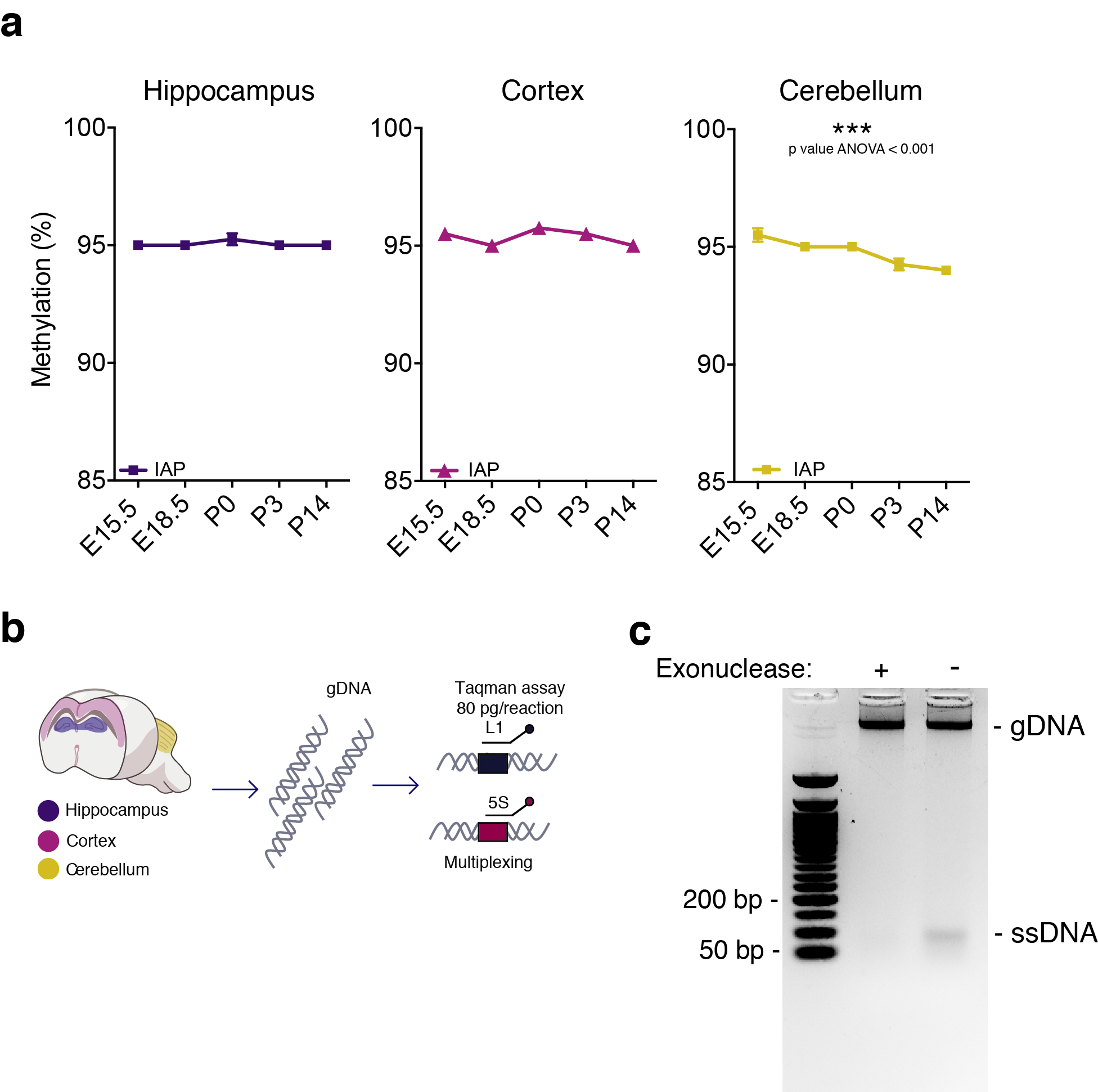


**Figure S3. IAP methylation levels do not change in hippocampus and cortex development while reduce in cerebellum development**

**a** Methylation analysis of four CpG sites on IAPLTR1a elements (as reported in (*34*)) in mouse hippocampus, cortex and cerebellum at different stages of embryonic (E15.5, E18.5) and postnatal (P0, P3 and P14) development. For each organ and developmental stage samples from the same 4 mice used in Fig. 4 were analyzed. Cerebellum: E15.5 vs P3, p < 0.001; E15.5 vs P14, p < 0.001; E18.5 vs P3, p = 0.0484; E18.5 vs P14, p = 0.007; P0 vs P3, p = 0.0484; P0 vs P14, p = 0.007, ANOVA with Tukey post hoc test. Data are represented as the mean percentage of methylation ± S.E.M. **b** Schematic representation of L1 CNV assay performed on mouse hippocampus, cortex and cerebellum. Briefly, the assay is performed in multiplex qPCR, using Taqman probes specific for mL1-ORF2 and m5S as reported in (*28*). **c** Control of exonuclease I treatment run on 2.2% agarose gel representing the efficiency of exonuclease I digestion of a single strand oligonucleotide of 120 bp.
