# Supplementary material for: Early maternal care restores LINE-1 methylation and enhances neurodevelopment in preterm infants
Source: BMC Med. 2021 Feb 5;19:42. doi: 10.1186/s12916-020-01896-0 (PMC7863536; doi:10.1186/s12916-020-01896-0)
Supplement: Supplementary file 2 — Additional file 2: Table S1. Baseline characteristics of the population: descriptive statistics and comparisons between Full-term and Preterm (both Early Intervention and Standard Care) groups. Table S2. Neurodevelopmental outcome at 12 months corrected age and 36 months chronological age of the overall population included in the RCT. Table S3. List of the primer sequences used in L1MdTf and IAPLTR1a NGS methylation analysis in mice. [file 12916_2020_1896_MOESM2_ESM.doc]

**Early maternal care restores LINE-1 methylation and enhances neurodevelopment in preterm infants**

Camilla Fontana ^1^†, Federica Marasca ^2^†, Livia Provitera ^3^, Sara Mancinelli ^4,5^, Nicola Pesenti ^3,6^, Shruti Sinha ^2^, Sofia Passera ^3^, Sergio Abrignani ^1,2^, Fabio Mosca ^1,3^, Simona Lodato ^4,5^, Beatrice Bodega ^2^‡*, Monica Fumagalli ^1,3^‡*.

^1^ University of Milan, Department of Clinical Sciences and Community Health, Milan, Italy.

^2^ Istituto Nazionale di Genetica Molecolare “Enrica e Romeo Invernizzi” (INGM), Milan, Italy.

^3^ Fondazione IRCCS Ca’ Granda Ospedale Maggiore Policlinico, NICU, Milan, Italy.

^4^ IRCCS Humanitas Clinical and Research Center, Rozzano - Milan, Italy.

^5^ Humanitas University, Department of Biomedical Sciences, Pieve Emanuele-Milan, Italy.

^6^ Department of Statistics and Quantitative Methods, Division of Biostatistics, Epidemiology and Public Health, University of Milano-Bicocca, Milan, Italy.

† Contributed equally as first author to this work

‡ Contributed equally as last author to this work.

* Correspondence should be addressed to: [monica.fumagalli@unimi.it](mailto:monica.fumagalli@unimi.it) and [bodega@ingm.org](mailto:bodega@ingm.orhg)

**Supplementary Tables**

**Additional File 2: Table S1-S3.**

**Table S1. Baseline characteristics of the population: descriptive statistics and comparisons between full-term and preterm (both early intervention and standard care) groups.**

**Table S2. Neurodevelopmental outcome at 12 months corrected age and 36 months chronological age of the overall population included in the RCT.**

**Table S3. List of the primer sequences used in L1MdTf and IAPLTR1a NGS methylation analysis in mice.**

| Demographic feature | Full-term  (n = 20) | Preterm  (n = 34) | p value |
| --- | --- | --- | --- |
| Gestational age at birth (weeks), mean ± SD | 38.5 ± 0.5 | 28.0 ± 1.2 | <0.001^ |
| Birth Weight (g), mean ± SD | 3234 ± 420 | 1045 ± 319 | <0.001^ |
| Male, n (%) | 6 (30%) | 18 (53%) | 0.156° |
| Cesarean Section, n (%) | 20 (100%) | 32 (94%) | 0.525° |
| Apgar score at 1’, median (range) | 9 (7-10) | 6 (2-8) | <0.001* |
| Apgar score at 5’, median (range) | 10 (8-10) | 8 (5-9) | <0.001* |
| Maternal Age (years), mean ± SD | 37.2 ± 3.8 | 34.0 ± 5.5 | 0.013^ |

**Table S1. Baseline characteristics of the population: descriptive statistics and comparisons between full-term and preterm (both early intervention and standard care) groups.**

Values are shown as count (percentage) for categorical variables and means ± standard deviations or median (range) for continuous variables. P – values were obtained using t – test (^), Mann – Whitney U test (*) or Fisher’s Exact Test (°).

|  | Standard  care | Early intervention | p value |
| --- | --- | --- | --- |
| 12 Months Follow-up | n=32 | n=29 |  |
| General Quotient, mean ± SD | 90.8 ± 4.1 | 93.5 ± 4.7 | 0.020^ |
| Locomotor, mean ± SD | 94.3 ± 6.4 | 93.1 ± 11.6 | 0.839* |
| Personal-Social, mean ± SD | 89.5 ± 5.8 | 93.6 ± 5.3 | 0.010* |
| Hearing and Language, mean ± SD | 91.1 ± 3.8 | 94.1 ± 3.7 | 0.011* |
| Eye and Hand Coordination, mean ± SD | 90.5 ± 6.1 | 94.7 ± 5.0 | 0.006^ |
| Performance, mean ± SD | 91.6 ± 5.7 | 94.7 ± 5.6 | 0.038^ |
|  |  |  |  |
| 36 Months Follow-up | n=24 | n=25 |  |
| General Quotient, mean ± SD | 85.4 ± 7.1 | 90.8 ± 4.2 | 0.003* |
| Locomotor, mean ± SD | 90.1 ± 4.8 | 92.0 ± 4.7 | 0.255* |
| Personal-Social, mean ± SD | 84.5 ± 6.0 | 89.7 ± 4.1 | 0.001^ |
| Hearing and Language, mean ± SD | 85.5 ± 10.1 | 92.1 ± 5.0 | 0.020* |
| Eye and Hand Coordination, mean ± SD | 87.1 ± 6.5 | 92.5 ± 4.5 | 0.002^ |
| Foundation of Learning ^a^, mean ± SD | 88.9 ± 5.3 | 93.1 ± 3.5 | 0.003^ |

**Table S2. Neurodevelopmental outcome at 12 months corrected age and 36 months chronological age of the overall population included in the RCT.**

Means ± standard deviations are shown. P-values were obtained using t-test (^) or Mann-Whitney U Test (*). ^a^ This subscale corresponds to “Performance” in GMDS-R

**Table S3. List of the primer sequences used in L1MdTf and IAPLTR1a NGS methylation analysis in mice.**

List of the primer sequences used in L1MdTf and IAPLTR1a methylation analysis in mice to unambiguously identify each organ at each developmental stage in the bisulfite sequencing analysis. Primer indexes are in bold.
